# Characteristics of Citizens and Their Use of Teleconsultations in Primary Care in the Catalan Public Health System Before and During the COVID-19 Pandemic: Retrospective Descriptive Cross-sectional Study

**DOI:** 10.2196/28629

**Published:** 2021-05-27

**Authors:** Oscar Solans, Josep Vidal-Alaball, Pasqual Roig Cabo, Núria Mora, Ermengol Coma, Josep Maria Bonet Simó, Eduardo Hermosilla Pérez, Francesc Saigí-Rubió, Carmen Olmos Domínguez, Jordi Piera-Jiménez, Mercè Abizanda González, Francesc López Seguí

**Affiliations:** 1 Health Department Catalan Ministry of Health Barcelona Spain; 2 Digitalization for the Sustainability of the Healthcare System Sistema de Salut de Catalunya Barcelona Spain; 3 Health Promotion in Rural Areas Research Group Gerència Territorial de la Catalunya Central Institut Català de la Salut Sant Fruitós de Bages Spain; 4 Unitat de Suport a la Recerca de la Catalunya Central Fundació Institut Universitari per a la Recerca a l'Atenció Primària de Salut Jordi Gol i Gurina Sant Fruitós de Bages Spain; 5 Faculty of Medicine University of Vic - Central University of Catalonia Vic Spain; 6 Northern Metropolitan Primary Care Directorate Institut Català de la Salut Badalona Spain; 7 Primary Care Services Information Systems Institut Català de la Salut Barcelona Spain; 8 Jordi Gol i Gurina Primary Health Care Research Institute Foundation Barcelona Spain; 9 Faculty of Health Sciences Universitat Oberta de Catalunya Barcelona Spain; 10 Interdisciplinary Research Group on ICTs Barcelona Spain; 11 Servei Català de la Salut Barcelona Spain; 12 Open Evidence Research Group Universitat Oberta de Catalunya Barcelona Spain; 13 Health Department eHealth Unit Barcelona Spain; 14 Pere Virgili Health Park Primary Care Management Control Barcelona Spain; 15 Centre de Recerca en Economia i Salut Pompeu Fabra University Barcelona Spain

**Keywords:** teleconsultation, primary care, remote consultation, telehealth, COVID-19, e-consultation

## Abstract

**Background:**

eConsulta—that is, asynchronous, two-way teleconsultation in primary care—is one of the most important telemedicine developments in the Catalan public health system, a service that has been heavily boosted by the onset of the COVID-19 pandemic. It is vital to know the characteristics of its users in order to be able to meet their needs and understand the coverage of this service in a context where there is reduced accessibility to the health system.

**Objective:**

This study aims to analyze the profile of the citizens who use the eConsulta tool and the reasons for their use, as well as to gain an understanding of the elements that characterize their decision to use it while distinguishing between those who used it before and those who have used it since the onset of the COVID-19 pandemic.

**Methods:**

A descriptive, observational study based on administrative data was performed. This study differentiates between the COVID-19 pandemic era and the period preceding it, considering the day the state of emergency was declared in Spain (ie, March 12, 2020) as the cut-off point. It also differentiates between eConsulta users who send messages and those who only receive them.

**Results:**

During the pandemic, the number of unique users of this teleconsultation service had almost tripled, with up to 33.10 visits per 1000 inhabitants per month reported in the first three months. For the two user profiles analyzed, most users since the start of the COVID-19 outbreak were predominantly female, systematically younger, more actively employed, and with less complex pathologies. Furthermore, eConsulta users received more messages proactively from the health professionals. There was also a relative decrease in the number of conversations initiated by higher-income urban users and an increase in conversations initiated by users in rural areas.

**Conclusions:**

The COVID-19 pandemic has helped to generalize the use of telemedicine as a tool to compensate, to some extent, for the decline in face-to-face visits, especially among younger citizens in Catalonia. Telemedicine has made it possible to maintain contact between citizens and the health care system in the context of maximum complexity.

## Introduction

The eConsulta tool, which has been in operation since 2015, is one of the most important telemedicine developments by the Catalan public health system. It is an asynchronous, two-way teleconsultation tool used by health professionals and citizens that is part of the patient portal of the public health system, a platform that also allows citizens to securely access their personal health information stored in the personal health folder and to carry out certain clinical and administrative procedures. This service was operational only in the context of primary care [1,2] until it was recently expanded to hospital care.

According to data from the Ministry of Health of Catalonia (email, March 3, 2021), from the inception of eConsulta in October 2015 up to February 22, 2021, a total of 15,569 primary care health professionals (out of about 19,000 with potential access) have carried out 4,263,665 e-consultations involving 1,061,995 citizens (out of a total population of 7.7 million. This service is a new model of care relating to health care professionals, more practical for users, and more efficient for the health system, in general [3,4]. In addition, it empowers citizens and promotes teleworking among health care professionals—an essential factor in the context of the current COVID-19 pandemic, which helps improve their work-life balance [5]. In recent years, even before the onset of the pandemic, the use of the eConsulta tool had grown significantly, among both citizens and health care professionals; however, this only represented a very small proportion in relation to the number of face-to-face visits conducted in the Catalan public primary care system [6], a situation very similar to that of other countries [7,8].

**Figure 1:**
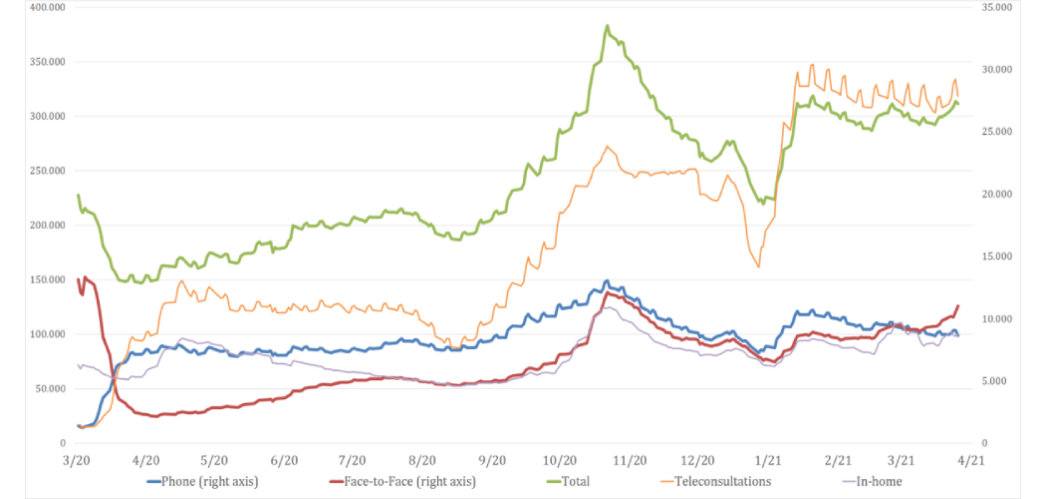
Primary care daily visits in the Catalonia region of Spain, classified by type with 7-day moving average excluding weekends and public holidays (March 2020 to April 2021). Source: Primary Care Services Information Systems, Institut Català de la Salut, Barcelona, Spain (SISAP) [7].

The onset of the COVID-19 pandemic and the initial need to reduce the risk of infection by preventing patients from physically visiting health centers has led to a change in the health care model that has promoted non–face-to-face care. The majority of countries have responded to this need by making significant efforts to implement both synchronous and asynchronous telemedicine approaches [9-12]. The demand for teleconsultation has considerably increased worldwide; for example, in France, the number of teleconsultations increased 50-fold during the initial weeks following the COVID-19 outbreak [13,14]. In Catalonia as well, the outbreak resulted in a very significant increase in the daily number of teleconsultations, although these numbers are well below the number of telephone consultations conducted (Figure 1).

Despite the fact that some studies question the efficiency of teleconsultations between primary care professionals and citizens [7], several studies suggest that the eConsulta tool helps to reduce the number of face-to-face visits [4,6], which may be one of the drivers of its growing use among users. With regard to the health care professionals that use eConsulta, a previous analysis found that doctors who use eConsulta are typically in the 45-64 years age group, score higher than the 80th percentile on the Quality of Care Index, have a high degree of accessibility, are involved in teaching, and work as part of a health team in a high socioeconomic urban setting [15]. With regard to eConsulta users, it is vital to analyze their characteristics in order to provide a service that is appropriate and adjusted to their needs and to also understand who is being covered by this service (and who is not) during the COVID-19 pandemic. Previous studies have shown that women use web-based consultations more frequently than men (64.7% vs 35.3%) and their average age is 39 years [7]. On the contrary, other studies have found that men send email communication for health care more often than women [16]. In Catalonia, however, there is no evidence to determine the profile of a typical citizen or their use of the eConsulta tool.

Thus, the aim of this study is to offer a descriptive analysis of the use of the eConsulta tool before and during the COVID-19 pandemic and the profile of the citizens who use it in order to gain a more comprehensive understanding of the elements that characterize their decision to use the tool and assess who is being covered through this service in a context of low accessibility to the health system.

## Methods

This is a descriptive, observational study based on administrative data sourced from the Catalan Health Institute (ICS), the main provider of primary care services in the Catalonia region, serving 74% of the Catalan population. The analysis period was from June 1, 2018, to June 15, 2020. Data of all living patients, as of December 2019 (N=5,844,804), assigned to an ICS primary care team during the study period were analyzed. All these citizens could access and use the eConsulta tool with prior authorization from a health care professional. The analysis differentiates between the COVID-19 pandemic era (ie, from March 12, 2020, the day the state of emergency was declared in Spain, to June 15, 2020) and the preceding period (ie, before March 12, 2020). It also differentiates between eConsulta users who send messages and those who only receive them.

The main study variable is the use of the eConsulta service. “Use” is defined as the period when any messages are sent between a health professional and a citizen, and “nonuse” is defined as the period when no messages have been sent during the study period. The following independent variables were considered in this study: age; gender; socioeconomic level of the center; type of center (rural or urban); adjusted morbidity group (GMA) indicator, a population grouping method that allows the population to be classified into excluding groups according to their multimorbidity [17]; a binary variable identifying the low-income immigrant population; patients with advanced chronic diseases (MACA) indicator [18]; patients with complex chronic diseases (PCC) indicator; and the level of pharmacy coverage. In addition, we assessed the socioeconomic status using the validated MEDEA (Mortality in small Spanish areas and Socioeconomic and Environmental Inequalities) deprivation index, which takes into account the variables of income, occupation, and level of education, among other factors [19]. We categorized this index into quartiles, with the 1st and 4th quartiles representing the least and most deprived areas, respectively. Rural areas were categorized separately and were defined as areas with less than 10,000 inhabitants and a population density lower than 150 inhabitants/km^2^.

Continuous variables are presented with mean and SD values, and variables with a nonnormal distribution are presented with median, minimum, and maximum values. Categorical variables are presented with the absolute and relative frequency of each category. For the comparison of two categorical variables, Fisher test and Chi-squared test were used; for the comparison of two numerical variables, the *t* test was used; and in cases where there were more than two variables, the analysis of variance (ANOVA) test was used. A significance level of 5% was set. Data were analyzed using R software (version 3.4.3; R Foundation for Statistical Computing).

## Results

Table 1 shows the characterization of citizens who used the service, grouped according to whether they have started a conversation or only received messages, during both the pre–COVID-19 and COVID-19 periods (*P*<.001). The results show that the profile of the typical user who started a conversation in the pre–COVID-19 era was female (33,096/56,494, 58.6%), with a mean age of 49.84 (17.06) years, a GMA of 2, residing mostly in an urban setting (45,977/56,494, 81.4%). Moreover, the user profile for those who only receive messages was found to be slightly older (mean age: 50.71 [SD 16.16] years), more male (8606/20,104, 42.8%), and more urban (16,492/20,104, 82.0%) compared with active users. When the user profile was analyzed according to the MEDEA deprivation index, we found that since the COVID-19 outbreak, there has been a decrease in the percentage of conversations initiated by higher-income urban citizens (from 4971/20,104, 24.7%, to 19,891/89,102, 22.3%) and an increase in the proportion of users from rural areas (from 10,517/56,494, 18.6%, to 26,958/130,941, 20.6%).

**Table 1:** Characteristics of citizens who use eConsulta (Source dataset: [20]).

Variable and period					eConsulta users (citizens)				
					Has initiated a conversation (Pre–COVID-19 period: N=56,494; COVID-19 period: N=130,941)		Nonuser (Pre–COVID-19 period: N=5,768,206; COVID-19 period: N=5,624,761)		Only receives messages (Pre–COVID-19 period: N=20,104; COVID-19 period: N=80,102)
**Number of messages, mean (SD)**									
	Pre–COVID-19 period			1.89 (0.39)		N/A^a^		1.06 (0.31)	
	COVID-19 period			1.70 (0.48)		N/A		1.02 (0.17)	
**Number of eConsultations, mean (SD)**									
	Pre–COVID-19 period			4.00 (5.08)		N/A		1.31 (0.75)	
	COVID-19 period			3.19 (3.05)		N/A		1.83 (1.43)	
**Age (years), mean (SD)**									
	Pre–COVID-19 period			49.84 (17.06)		42.67 (23.12)		50.71 (16.16)	
	COVID-19 period			44.96 (20.04)		42.68 (23.22)		45.02 (15.82)	
**Gender (female), n (%)**									
	Pre–COVID-19 period			33,096 (58.58)		2,920,933 (50.64)		11,498 (57.19)	
	COVID-19 period			75,892 (57.96)		2,839,296 (50.48)		50,339 (56.50)	
**GMA^b^, n (%)**									
		**1**							
			Pre–COVID-19 period	14,164 (25.07)		2,851,985 (49.44)		4597 (22.87)	
			COVID-19 period	35,139 (26.84)		2,810,872 (49.97)		24,735 (27.76)	
		**2**							
			Pre–COVID-19 period	24,033 (42.54)		1,697,448 (29.43)		8519 (42.37)	
			COVID-19 period	56,896 (43.45)		1,632,369 (29.02)		40,735 (45.72)	
		**3**							
			Pre–COVID-19 period	13,382 (23.69)		850,893 (14.75)		5317 (26.45)	
			COVID-19 period	29,522 (22.55)		820,537 (14.59)		19,533 (21.92)	
		**4**							
			Pre–COVID-19 period	4475 (7.92)		285,988 (4.96)		1548 (7.70)	
			COVID-19 period	8760 (6.69)		279,505 (4.97)		3746 (4.20)	
**Immigrant, n (%)**									
	Pre–COVID-19 period			1369 (2.42)		789,383 (13.69)		642 (3.19)	
	COVID-19 period			4836 (3.69)		779,631 (13.86)		6927 (7.77)	
**MACA^c^, n (%)**									
	Pre–COVID-19 period			186 (0.33)		13,167 (0.23)		46 (0.23)	
	COVID-19 period			348 (0.27)		12,886 (0.23)		165 (0.19)	
**MEDEA^d^, n (%)**									
		**0R^e^**							
			Pre–COVID-19 period	3222 (5.70)		363,932 (6.31)		1146 (5.70)	
			COVID-19 period	6001 (4.58)		357,023 (6.35)		5276 (5.92)	
		**1R^f^**							
			Pre–COVID-19 period	2306 (4.08)		331,292 (5.74)		624 (3.10)	
			COVID-19 period	5965 (4.56)		323,592 (5.75)		4665 (5.24)	
		**2R^g^**							
			Pre–COVID-19 period	4989 (8.83)		693,257 (12.02)		1842 (9.16)	
			COVID-19 period	14,992 (11.45)		672,972 (11.96)		12,124 (13.61)	
		**1U^h^**							
			Pre–COVID-19 period	18,273 (32.35)		1,233,124 (21.38)		4971 (24.73)	
			COVID-19 period	35,771 (27.32)		1,200,706 (21.35)		19,891 (22.32)	
		**2U^i^**							
			Pre–COVID-19 period	7168 (12.69)		864,044 (14.98)		2906 (14.45)	
			COVID-19 period	18,879 (14.42)		842,841 (14.98)		12,398 (13.91)	
		**3U^j^**							
			Pre–COVID-19 period	13,513 (23.92)		1,190,933 (20.65)		5265 (26.19)	
			COVID-19 period	31,120 (23.77)		1,159,874 (20.62)		18,717 (21.01)	
		**4U^k^**							
			Pre–COVID-19 period	7023 (12.43)		1,091,624 (18.92)		3350 (16.66)	
			COVID-19 period	18,213 (13.91)		1,067,753 (18.98)		16,031 (17.99)	
**Level of coverage, n (%)**									
		**Active**							
			Pre–COVID-19 period	44,581 (78.91)		4,262,326 (73.89)		15,268 (75.95)	
			COVID-19 period	108,917 (83.18)		4,133,267 (73.48)		79,991 (89.77)	
		**Pensioner**							
			Pre–COVID-19 period	10,890 (19.28)		1,143,779 (19.83)		4311 (21.44)	
			COVID-19 period	19,233 (14.69)		1,132,752 (20.14)		6995 (7.85%)	
**PCC^l^**									
	Pre–COVID-19 period			1695 (3.0)		103,214 (1.79)		459 (2.28)	
	COVID-19 period			2878 (2.2)		101,411 (1.80)		1079 (1.21)	
**Rural residents, n (%)**									
	Pre–COVID-19 period			10,517 (18.62)		1,388,481 (24.07)		3612 (17.97)	
	COVID-19 period			26,958 (20.59)		1,353,587 (24.06)		22,065 (24.76)	

^a^N/A: not applicable.

^b^GMA: adjusted morbidity group.

^c^MACA: patients with advanced chronic diseases.

^d^MEDEA: mortality in small Spanish areas and socioeconomic and environmental inequalities

**^e^**0R: Rural.

**^f^**1R: Semirural.

**^g^**2R: Semiurban.

**^h^**1U: Urban, very high socioeconomic level.

**^i^**2U: Urban, high socioeconomic level.

**^j^**3U: Urban, low socioeconomic level.

**^k^**4U: Urban, very low socioeconomic level.

^l^PCC: patients with complex chronic diseases.

Since the start of the COVID-19 outbreak, eConsulta users have been predominantly female. However, users during the COVID-19 period were systematically younger than those before the pandemic, for the two user profiles analyzed. An increase was observed in the percentage of eConsulta use (especially with regard to the number of messages received from health care professionals) in those population profiles that are more actively employed and have fewer chronic diseases. Thus, we observed a group of working-age citizens who did not used to go to the doctor and citizens who, when they have had to go, preferred the non–face-to-face channel.

In addition, the analysis of the volume of use of the tool during the pre–COVID-19 and COVID-19 periods shows that the number of unique users almost tripled in the first 3 months of the pandemic (from 76,598 to 220,043, 2.87% increase; Table 2). The number of consultations in the 3 months before and after the pandemic showed a monthly increase from 5.61 to 33.10 visits per 1000 inhabitants.

Of these consultations, the proportion of conversations initiated by citizens had reduced (from 74.95% to 52.48%), whereas those initiated by professionals had almost doubled (from 25.05% to 47.52%). Furthermore, the proportion of messages sent by professionals had substantially increased. Similarly, the proportion of conversations involving a response from the professional had decreased considerably (from 1.79% to 0.47%), and the proportion of conversations involving a response initiated by the citizen (ie, patient) had slightly reduced (from 90.8% to 80.61%), as shown in Table 3.

**Figure 2:**
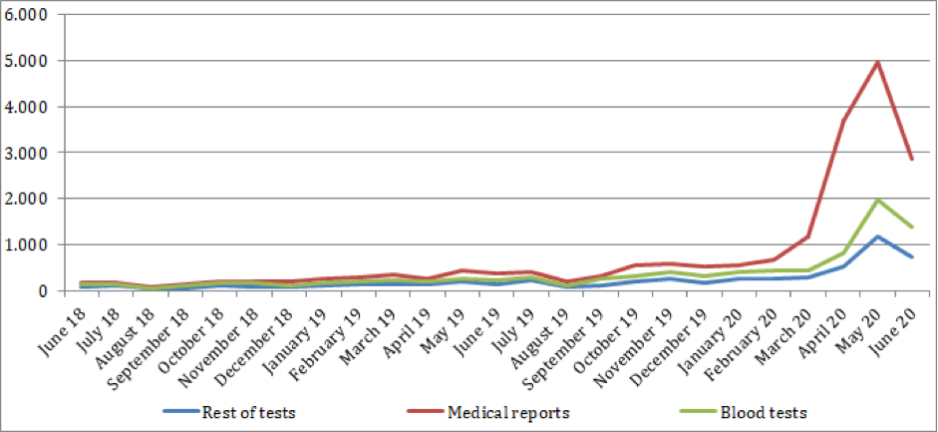
Evolution of the number of documents attached to eConsulta messages, classified by type.

The number of documents attached to eConsulta messages sent by citizens before and during the COVID-19 pandemic was also analyzed (Table 4 and Figure 2). A clear increase in the number of files sent, especially in terms of medical reports, was observed.

Finally, the variations in overall, face-to-face, and web-based care received by users of different age groups were analyzed. Table 5 shows that the use of telemedicine has somewhat mitigated the decline in face-to-face visits in younger age groups.

**Table 2:** eConsulta activity before and after the COVID-19 pandemic.

Period of analysis	Users (citizens), n (%) (N= 5,844,804)	Consultations, n
Pre–COVID-19 period (June 1, 2018, to March 12, 2020)	76,598 (1.31)	252,370
COVID-19 period (March 13, 2020, to June 15, 2020)	220,043 (3.76)	580,496

**Table 3:** Activity by initiator (health care professional or citizen).

Initiator and period		Value, n (%)		
		Number of conversations initiated (Pre–COVID-19 period: N=252,370; COVID-19 period: N=580,496)	Number of conversations with a response	Number of messages sent (Pre–COVID-19 period: N=441,656; COVID-19 period: N=863,867)
**Citizens**				
	Pre–COVID-19 period	189,145 (74.95)	175,534 (92.80)	195,219 (44.20)
	COVID-19 period	304,639 (52.48)	245,582 (80.61)	315,656 (36.54)
**Health care professionals**				
	Pre–COVID-19 period	63,225 (25.05)	1,134 (1.79)	246,437 (55.80)
	COVID-19 period	275,857 (47.52)	1,286 (0.47)	548,211 (63.46)

**Table 4:** Average number of monthly tests, classified by type.

Test	Value, mean (SD)		*t* test (*df*)	*P* value
	Pre–COVID-19 period	COVID-19 period		
Medical reports	328.05 (168.02)	3179.50 (1590.69)	3.58 (3.01)	.04
Blood tests	223.62 (107.12)	1151.75 (669.03)	2.77 (3.02)	.07
Other tests	140.57 (64.92)	672.75 (37.01)	2.81 (3.03)	.07

**Table 5:** Number of face-to-face visits and teleconsultations, classified by age group. Source: Primary Care Services Information Systems, Institut Català de la Salut, Barcelona, Spain (SISAP) [21].

Visit type and age (years)		March to June 2019	March to June 2020	Year-on-year variation (%)
**Overall**				
	<16	28,824	34,722	20.5
	16-44	1,756,336	1,786,902	1.7
	45-64	2,106,944	2,065,822	–2.0
	65-74	1,053,413	706,222	–33.0
	>75	1,486,168	1,094,727	–26.3
**eConsulta visit** **, n (%)**				
	<16	16 (0.06)	550 (1.58)	3337.5
	16-44	14,155 (0.81)	173,647 (9.72)	1126.8
	45-64	18,072 (0.86)	180,448 (8.73)	898.5
	65-74	4,044 (0.38)	16,702 (2.36)	313.0
	>75	4,863 (0.33)	16,793 (1.53)	245.3
**Face-to-face visit** **, n (%)**				
	<16 years	26,188 (90.85)	11,501 (33.12)	–56.1
	16-44	1,424,661 (81.12)	427,756 (23.94)	–70.0
	45-64	1,614,686 (76.64)	471,869 (22.84)	–70.8
	65-74	786,859 (74.7)	200,202 (28.35)	–74.6
	>75	1,020,346 (68.66)	316,402 (28.9)	–69.0

## Discussion

### Principal Findings

The COVID-19 outbreak has led to a significant increase in the use of teleconsultation by both citizens of the Catalonia region and health care professionals. The number of e-consultations per 1000 inhabitants has increased from 5.61, a figure that can be considered low compared to other studies [7], to 33.10 after the start of the COVID-19 pandemic. This increase is explained, first, by the fact that before the onset of the pandemic, patients needed to receive an authorization from their health care professional to be able to carry out e-consultations. After the COVID-19 outbreak, this permission was extended to all citizens. The greater use of teleconsultation is also explained by the increase in the remote provision of care processes that were performed in-person before the pandemic, enabling these citizens to receive remote assistance (ie, sick leave), remote updating of electronic prescription plans for chronic patients, or reactive prescriptions for non–face-to-face visits for acute patients and guidelines for monitoring oral anticoagulant use in patients receiving treatment, among others). The reduction in the average age and the 5-fold increase in “passive” users (ie, users who receive but do not send messages) suggest that eConsulta has been widely used for notifying results or sick leave(s) by allowing users to connect to their personal health folder.

The pandemic has modulated the way eConsulta is used and the typical user profile. There has been an increase in use by both health care professionals, with a clear increase in their initiative in sending messages and documents to citizens, and young patients without chronic diseases. This group comprises citizens, who before the pandemic were infrequent users of the system, but because of the pandemic and their acute pathologies—often related to COVID-19—have had to use the tool in order to contact the health services or receive test results. These processes have made it possible to continue to offer key and prevalent primary care processes in a non–face-to-face and safe and stable manner, by avoiding visits to health centers and contributing decisively to reducing the risk of infection during periods of strict lockdown. It should be noted that eConsulta has not been used for the purpose of COVID-19–related mass messaging by the health system and that it has only been used for the purpose of care continuity for patients.

The results of this study show that it is necessary to understand the type of use of the tool in order to make improvements in its operation and continue working on a model that improves the management of demand for primary care and, in return, its efficiency. To date, the approach based on free-text analysis using machine learning tools seems to be a suitable option to study the evolution over time of the use of the teleconsultation service [3]. This, however, would be a suboptimal solution. The planned evolution of the eConsulta tool for the coming months is precisely the structured stratification of the reasons for consultation, reported by the citizens themselves before initiating it, which will initially allow these messages to be redirected to the professional profiles (doctor, nurse, administrator, dentist, social worker, etc) that can respond in a more agile and appropriate way based on the need expressed by the citizen in their message. In this way, care is decentralized to the different professional profiles, allowing a more efficient response to the citizen based on the reason for their consultation.

The pandemic caused by the novel coronavirus SARS-CoV-2 has changed the model of health care, and this is especially noticeable in primary care centers, where the most commonly used face-to-face model has been replaced by a mixed model in which telemedicine tools play a very significant role. Although it is clear that the pandemic has led to a reduction in the diagnosis of many diseases [22] and the control of chronic diseases [23], it is necessary to assess the effects of these changes in care (forced by the circumstances) on the health of the supported population in order to continue to guarantee quality health care. We need to emerge from this crisis with a clearer vision of how to continue to deploy telemedicine to obtain its benefits by avoiding or minimizing its drawbacks [12] and inequalities regarding access for the most vulnerable groups [24].

The acceleration of digital transformation processes in health centers has ensured the continuity of care of many basic care processes for the population during lockdown [11]. This new model of care is changing the way we interact with the health care system and the patient profile used by each channel. The results of this study show that non–face-to-face communications from the Catalan primary health care system are being used predominantly in favor of the low-risk and younger population and, therefore, preserving face-to-face and home visits for the most complex and older populations. This adaptation and flexibility of the health system’s response, based on the different needs and types of patients, is beneficial and demonstrates the resilience that telemedicine tools have provided to the health system during the COVID-19 pandemic.

This study presents some limitations, as it does not evaluate the relationship between unanswered e-consultations and the type of visit, either in person or by telephone for the same user. It could be possible that the objective of many citizens who have sent an e-consultation was actually to receive a face-to-face or telephone visit in which case the e-consultation would not have replaced these other types of visits.

### Conclusions

In the context of reduced face-to-face accessibility to the health system, this study has highlighted a change in the profile of the Catalan citizen using the eConsulta telemedicine tool. Since the start of the COVID-19 pandemic, this patient or user profile is similar to that of the average citizen: actively employed, with less complex pathologies, and who receives more messages proactively from health care professionals through this tool. The COVID-19 pandemic has helped to socialize the use of telemedicine, and as a result, it has helped to mitigate, to some extent, the decline in face-to-face visits in younger age groups.

## Abbreviations

ANOVA: analysis of variance
ICS: Catalan Health Institute
GMA: adjusted morbidity group
MACA: patients with advanced chronic diseases
MEDEA: Mortality in small Spanish areas and Socioeconomic and Environmental Inequalities
PCC: patients with complex chronic diseases
